# Preparation of Polysilsesquioxane-Based RO Membranes with Urea Units for Water Desalination

**DOI:** 10.3390/membranes15100322

**Published:** 2025-10-20

**Authors:** Joji Ohshita, Katsuhiro Horata, Toshiki Kaneko, Yohei Adachi, Masakoto Kanezashi

**Affiliations:** 1 Smart Innovation Program, Graduate School of Advanced Science and Engineering, Hiroshima University, Higashi-Hiroshima 739-8527, Japan; 2 Division of Materials Model-Based Research, Digital Monozukuri (Manufacturing) Education and Research Center, Hiroshima University, Higashi-Hiroshima 739-0046, Japan; 3 Chemical Engineering Program, Graduate School of Advanced Science and Engineering, Hiroshima University, Higashi-Hiroshima 739-8527, Japan

**Keywords:** polysilsesquioxane, reverse osmosis membrane, water desalination

## Abstract

Seawater and brackish water desalination using membranes is anticipated to offer a simple and effective solution to the global water shortage, and polysilsesquioxane (PSQ) is expected to be the base material for robust reverse osmosis (RO) membranes for water desalination. Hydroxyethylurea-containing PSQ-based RO membranes for water desalination have recently been developed via a sol–gel process. Although these membranes showed high performance, achieving a water permeability of 1.86 × 10^−12^ m^3^ m^−2^s^−1^Pa^−1^ and an NaCl rejection of 95.9%, the membranes showed limited chlorine resistance and processibility and moderate heat resistance. In this study, three new urea-containing monomers were designed and prepared for RO membrane preparation. The copolymerization of these urea-containing monomer with bis(triethoxysilylpropyl)amine resulted in performance comparable to that of hydroxyethylurea-containing PSQ membranes. The present urea-containing PSQ membranes exhibited enhanced chlorine resistance, with only 1–3% decreases in NaCl rejection, even after 10,000 ppm h exposure to chlorine, together with 3–19% increases in water permeability. Additionally, the presently prepared urea-containing PSQ membranes exhibited improved processability. This study provides a new molecular design for robust and high-performance RO membranes that can be prepared through a simple sol–gel process.

## 1. Introduction

Water shortage is a global problem that has a serious impact on human life in large areas of the world. Desalination of seawater and brackish water using membranes is anticipated to offer a simple and effective solution to this problem [1]. Membrane separation requires less energy than conventional separation methods, such as distillation, making it more economical. Generally, organic polymer membranes, typically aromatic polyamide membranes, are used as reverse osmosis (RO) membranes for water desalination [2,3,4,5,6]. However, these membranes typically exhibit limited thermal and chlorine resistance, reducing their lifespan. Numerous studies have been conducted on the modification of polyamide membranes, such as composite formation with graphene oxide, to improve their stability [7,8,9,10,11,12,13,14,15,16]. Polysilsesquioxanes (PSQs) have been extensively studied as organic–inorganic hybrid materials that can be readily prepared by the hydrolysis/condensation of trifunctional silanes, typically trialkoxysilanes (Equation (1)) [17,18,19,20]. PSQs are anticipated to possess both advantageous properties, such as high thermal and mechanical stability, and solubility and processability, attributed to the inorganic siloxane (Si-O-Si) bond framework and organic substituents (R) on silicon atoms, respectively. During the studies on PSQ-based materials, PSQ-based RO membranes were developed for water desalination. Although these membranes exhibit high stability owing to the fundamental characteristics of PSQs based on an inorganic framework, they usually exhibit low water permeability, hindering their practical applications [21].

RSi(OEt)_3_ → (RSiO_1.5_)*_n_* (1)

**Figure 1:**
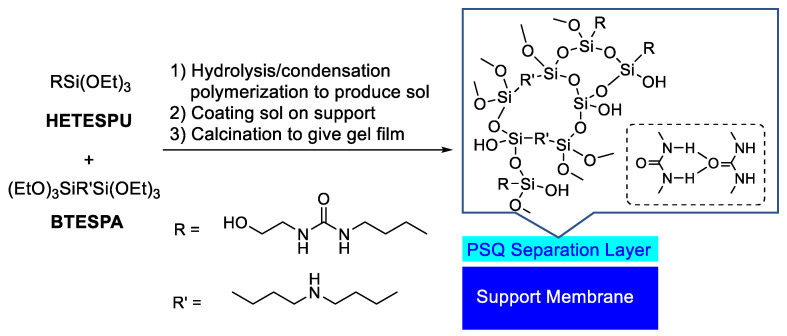
Fabrication of HETESUP-BTESPA PSQ copolymer membrane and hydrogen bonding expected for urea-urea interaction (dotted box).

Generally, two important parameters of desalination RO membranes, NaCl rejection and water permeability, are in a trade-off relationship, making it difficult to enhance both simultaneously. Recently, hydroxy groups were introduced to PSQ membranes to improve their hydrophilicity and, consequently, their water permeability without significantly affecting NaCl rejection [22,23,24]. Similarly, Yamamoto, Gunji, and coworkers reported the utilization of cage-type PSQ oligomers with carboxylic acid units as monomers for PSQ-based RO membranes [25,26]. The modification of an ethenylene-bridged PSQ RO membrane with 2-mercaptosuccinic acid via an ene-thiol reaction to enhance water permeability has also been reported [27]. Among the hydroxy-containing membranes, the hydroxyethylurea-containing PSQ membrane prepared by the copolymerization of *N*-hydroxyethyl-*N*’-(triethoxysilylpropyl)urea (HETESPU) and bis(triethoxysilylpropyl)amine (BTESPA) demonstrated superior performance, with a water permeability of 1.86 × 10^−12^ m^3^ m^−2^s^−1^Pa^−1^ and an NaCl rejection of 95.9% when the monomer ratio of HETESPU:BTESPA was 1:3 (Figure 1) [24]. The improved permeability is attributable to the formation of hydrophilic channels through the aggregation of hydroxyethylurea units via hydrogen bonding. Hydrogen bonding between urea units in PSQ materials has been previously reported [28,29]. These data are comparable to those of a typical commercial aromatic polyamide membrane (SW30HR: water permeability = 1.1 × 10^−12^ m^3^ m^−2^s^−1^Pa^−1^ and NaCl rejection = 98.5%) [30]. However, the HETESPU-BTESPA RO membranes were found to be less stable upon exposure to chlorine than other PSQ-based RO membranes, and exposure to an NaOCl aqueous solution for 10,000 ppm h resulted in a decrease in the NaCl rejection to 76.0%, although the water permeability did not change significantly. The performance of the HETESPU-BTESPA RO membranes was significantly affected by the monomer ratio, and excessive amounts of HETESPU resulted in a decrease in performance due to over-aggregation, which induced defect formation on the membrane surface. Therefore, the monomer ratio must be precisely controlled to prepare high-performance HETESPU-BTESPA RO membranes.

**Figure 2:**
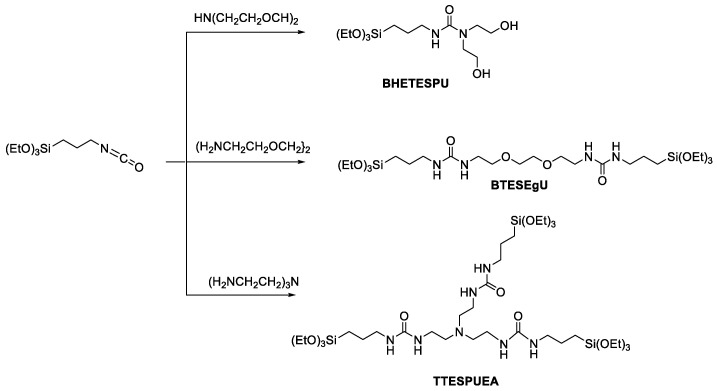
Synthesis of urea-containing triethoxysilyl monomers.

In this study, three new monomers were prepared (Figure 2). The copolymerization of these urea-containing monomers with BTESPA provided RO membranes applicable to water desalination, exhibiting markedly enhanced chlorine resistance compared to HETESPU-BTESPA membranes. Although the (3,6-dioxaoctane-1,8-diyl)bis-*N*-[*N*’-(triethoxysillylpropyl)urea] (BTESEgU)-BTESPA and (triethylamine-2,2′,2″-triyl)tris-*N*-[*N*’-(triethoxysillylpropyl)urea] (TTESPUEA)-BTESPA membranes showed the formation of indentations on the surface, the *N,N*-bis(hydroxyethyl)-*N*’-(triethoxysilylpropyl)urea (BHETESPU)-BTESPA, BTESEgU-BTESPA, and TTESPUEA-BTESPA membranes showed improved processability, providing good performance as RO membranes over a wide range of monomer ratios.

## 2. Experimental

### 2.1. Materials and Methods

BTESPA was purchased from Gelest Inc. (Morrisville, NC, USA) and used as received. Other starting compounds were purchased from Tokyo Chemical Industry Co., Ltd. (Tokyo, Japan). All reactions were performed under a dry argon atmosphere. Dichloromethane, which was used as the reaction solvent, was distilled from P_2_O_5_ and stored on activated molecular sieves until use. NMR spectra were recorded on Varian Inc. (Palo Alto, CA, USA) 400 and 500 spectrometers. APCI MS spectra were obtained using a Thermo Fisher Scientific Inc. (Waltham, MA, USA) LTQ Orbitrap XL spectrometer at N-BARD, Hiroshima University, Japan. Fourier-transform infrared (FTIR) spectra were recorded using a Shimadzu Corp. (Kyoto, Japan) IR Affinity-1 spectrometer equipped with an Attenuated Total Reflectance (ATR) unit. Ethanol used for the sol–gel reactions was distilled from Mg(OEt)_2_ and stored on activated molecular sieves until use. Nitto Denko Corp. (Osaka, Japan) NTR-7450 was used as the support membrane (pore size: 1 nm), and its surface was gently washed with ethanol and dried at 60 °C immediately before use. Dynamic light scattering (DLS) analysis was performed using a Malvern Panalytical (Worcestershire, UK) ZEN3600 analyzer. The electric conductivities of the aqueous solutions eluted through the membranes were determined using a HORIBA, Ltd. (Kyoto, Japan) ES-51 conductivity meter. Scanning electron microscopy (SEM) was performed using a Hitachi High-Tech Corp. (Tokyo, Japan) S-4800 microscope for Pt-coated samples.

### 2.2. Preparation of BHETESPU

To a solution of diethanolamine (792 mg, 3.2 mmol) in 10 mL of dichloromethane and 2 mL of ethanol, triethoxysilylpropyl isocyanate (349 mg, 3.32 mmol) was added slowly, and the resulting mixture was stirred at room temperature for 1 h. The solvent was removed under reduced pressure to obtain 1.13 g (100% yield) of BHETESPU as a colorless viscous oil. NMR analysis of the oil revealed sufficient purity, and the oil was used for sol formation without further purification. IR 3330 (NH and OH), 1620 (C=O), 1540 (N-H) cm^−1^. ^1^H NMR (400 MHz in CD_3_OD) δ 3.82 (q, 6H, *J* = 7.0 Hz), 3.68 (t, 4H, J = 5.4 Hz), 3.42 (t, 4H, *J* = 5.4 Hz), 3.12 (t, 2H, *J* = 7.0 Hz), 1.54–1.63 (m, 2H), 1.21 (t, 9H, *J* = 7.0 Hz), 0.59–0.65 (m, 2H); ^13^C NMR (126 MHz in CD_3_OD) δ 161.38, 61.98, 59.37, 52.01, 44.32, 24.53, 18.66, 8.45; MS 353.21 (M^+^).

### 2.3. Preparation of BTESEgU

To a solution of 1,2-bis(2-aminoethoxy)ethane (474 mg, 3.2 mmol) in 10 mL of dichloromethane, triethoxysilylpropyl isocyanate (1.58 g, 6.4 mmol) was added slowly, and the resulting mixture was stirred at room temperature for 6 h. The solvent was removed under reduced pressure to obtain 2.06 g (100% yield) of BTESEgU as a white solid. NMR analysis of the solid revealed sufficient purity, and it was used without further purification. IR 3320 (NH), 1620 (C=O), 1580 (N-H) cm^−1^; ^1^H NMR (400 MHz in CDCl_3_) δ 5.17–5.29 (m, 4H), 3.81 (q, 12H, J = 7.0 Hz), 3.61 (s, 4H), 3.58 (t, 4H, *J* = 5.1 Hz), 3.34 (q, 4H, *J* = 5.1 Hz), 3.17 (q, 4H, *J* = 6.3 Hz), 1.51–1.67 (m, 2H), 1.22 (t, 18H, *J* = 7.0 Hz), 0.59–0.69 (m, 4H); ^13^C NMR (126 MHz in CDCl_3_) δ 159.04, 70.82, 70.41, 58.53, 42.97, 40.53, 23.77, 18.42, 7.76; MS 643.38 (M^+^).

### 2.4. Preparation of TTESPUEA

To a solution of tris(2-aminoethyl)amine (468 mg, 3.2 mmol) in 10 mL of dichloromethane, triethoxysilylpropyl isocyanate (2.37 g, 9.6 mmol) was added slowly, and the resulting mixture was stirred at room temperature for 1 h. The solvent was removed under reduced pressure to yield 2.84 g (100% yield) of TTESPUEA as a colorless solid. NMR analysis of the solid revealed sufficient purity, and it was used without further purification. IR 3330 (NH), 1620 (C=O), 1580 (N-H) cm^−1^; ^1^H NMR (400 MHz in DMSO-d_6_) δ 5.93 (t, 3H, *J* = 5.8 Hz), 5.78 (t, 3H, *J* = 5.5 Hz), 3.73 (q, 18H, *J* = 7.0 Hz), 2.90–3.04 (m, 12H), 2.42 (t, 6H, *J* = 6.3 Hz), 1.35–1.45 (m, 6H), 1.14 (t, 27H, *J* = 7.0 Hz), 0.47–0.54 (m, 6H); ^13^C NMR (126 MHz in DMSO-d_6_) δ 158.19, 57.67, 54.44, 42.08, 37.73, 23.57, 18.18, 7.28; MS 888.53 (M^+^).

### 2.5. Sol–Gel Process

The sol–gel reaction was performed as described previously [31]. The monomers in a specific ratio with H_2_O were stirred in ethanol at room temperature until the sol particle sizes become 1.5–10 nm (Table 1). The resultant sol was diluted with ethanol to 0.5–5 wt% based on the monomer weight, and the sol was kept in a tightly sealed glass vial at 4 °C until use.

### 2.6. Membrane Preparation

Membrane preparation was performed as previously described [24]. The sol was poured onto a support membrane (pore size: 1 nm) at room temperature. After 30 s, the excess sol was removed by decantation, and the membrane was air-dried at room temperature. This coating process was repeated twice, and the resultant membrane was calcined at 150 °C for 10 min in air.

### 2.7. Evaluation of RO Performance

**Figure 3:**
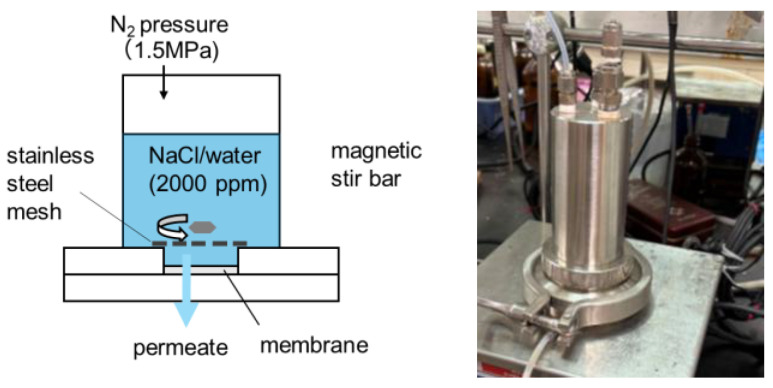
Schematic of the device structure and its photograph used for RO experiments.

The RO performance was evaluated as described previously, utilizing the device depicted in Figure 3 [25]. The device was filled with a 2000 ppm NaCl aqueous solution, and a feed pressure of 1.5 MPa was applied to the device at 25 °C. The volume and electrical conductivity of the permeated water were measured to determine the water permeability (*L_p_*) and NaCl rejection (*R*) using the following equations (Equations (2) and (3)), where *J_v_* is water flux, Δ*P* and Δ*π* are differences in applied and osmotic pressures, and *C_p_* and *C_f_* are NaCl concentrations in the permeated and feed solutions, respectively.

*L*_*p*_ = *J*_*v*_/(Δ*P*−Δ*π*) (2)

R = (1−*C*_*p*_/*C*_*f*_) (3)

## 3. Results and Discussion

### 3.1. Membrane Preparation

**Figure 4:**
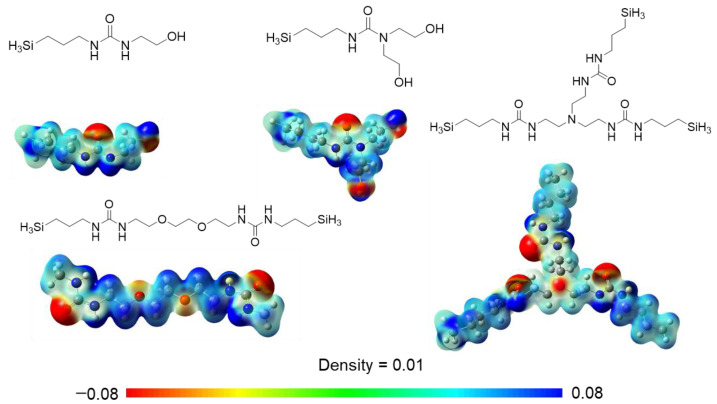
ESP maps derived from DFT calculations performed on the urea-containing monomer models at the B3LYP/6-31G level of theory using Gaussian 09. The urea and hydroxy units, as well as the ethylene glycol and amine units, play a role in enhancing the molecular polarity.

Three new monomers were prepared via simple one-step reactions (Figure 2) for RO membrane preparation with enhanced chlorine resistance and processability. It has been reported that urea degradation by chlorine is triggered by the replacement of NH with NCl [32]. BHETESPU was designed to possess fewer NH bonds than HETESPU, which are reactive towards chlorine. In contrast, BTESEgU and TTESPUEA have two and three triethoxysilyl units, respectively, leading to enhanced network structures in their PSQ films, improving chlorine resistance. In addition, BHETESPU, which has two hydroxyethyl groups on the urea nitrogen atom, should have higher hydrophilicity and flexibility than HETESPU, which enhances its water permeability and processability. BTESEgU and TTESUEA contain flexible and polar ethylene glycol and triethylamine linkers, respectively, in their structures, which also increase the water permeability and processability of the membranes. Indeed, DFT calculations suggested that the urea and hydroxy units, as well as the ethylene glycol and amine units, contribute to the enhancement of molecular polarity. As shown in Figure 4, electrostatic potential (ESP) calculations were performed on the model compounds of the monomers. The ESP maps indicate that the oxygen and nitrogen atoms of the ethylene glycol and amine units are negatively charged, although this is not evident compared to the urea carbonyl oxygens.

**Figure 5:**
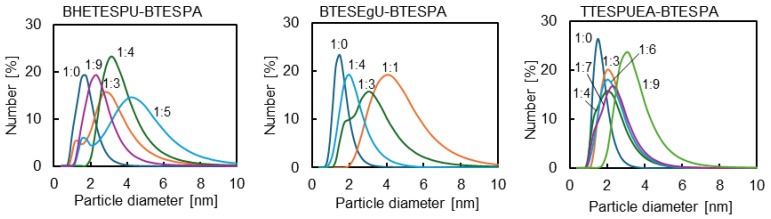
Particle size distributions of urea-containing PSQ sols determined by DLS.

**Figure 6:**
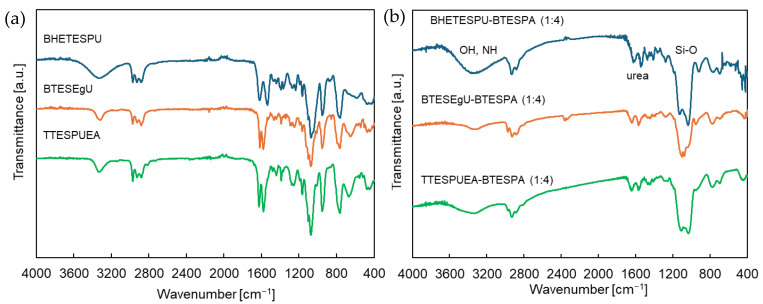
IR spectra of urea-containing (**a**) monomers and (**b**) PSQ films calcined at 150 °C in air on KBr plates.

These monomers were hydrolyzed with water in ethanol to obtain sols with appropriate particle size of 1.5–10 nm as presented in Table 1 and Figure 5. The copolymerization of the urea-containing monomers with BTESPA proceeded more rapidly than homopolymerization, which was attributed to the amine site of BTESPA catalyzing the polymerization. The sol was then coated onto an organic support membrane and calcined at 150 °C in air to promote gelation. The IR spectra of the gel film prepared by coating a KBr plate with the sol and calcination at the same temperature are shown in Figure 6. The spectra showed broad bands around 3300 cm^−1^, corresponding to the stretching frequencies of the O-H and N-H bonds. The broad Si-O stretching bands at approximately 1000 cm^−1^ were intensified compared to those of the monomers. For BHETESPU-BTESPA and TTESPUEA-BTESPA, the stretching bands of the C-O bonds likely overlapped with those of the Si-O bonds. The BHETESPU-BTESPA containing C-OH units showed a more pronounced O-H/N-H band than the others. The spectra also revealed two sharp bands at 1620–1650 and 1540–1570 cm^−1^. These bands are characteristic of urea units bearing interunit hydrogen bonding [33], indicating that the urea units did not decompose during calcination (Figure 6). Although the thermal decomposition of urea has been reported to begin at 152 °C [34], it was previously reported that monosubstituted urea units in PSQ decompose at higher temperatures [35].

### 3.2. RO Performance

**Figure 7:**
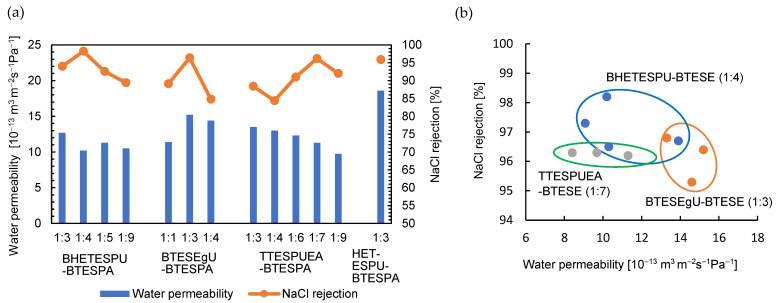
Summary of RO experiments of urea-containing PSQ membranes. (**a**) Effect of monomer ratio and (**b**) data variability for membranes with optimal performance.

RO experiments were conducted at room temperature. Generally, water permeability and NaCl rejection are improved with increasing operation time. This enhancement is attributed to the permeation of NaCl-containing water into the membrane, which enhances the membrane wettability and induces electrostatic repulsion between the salts absorbed in the membrane and those dissolved in the aqueous phase. The permeability and NaCl rejection were recorded after 1 h and subsequently at 2 h intervals, and the data that became stable after three to five hours of operation time are summarized in Table 2 and Figure 7.

**Figure 8:**
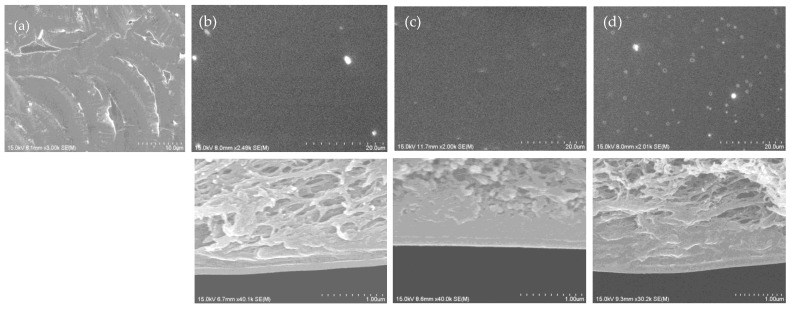
Surface (**top**) and cross-section (**bottom**) SEM images of (**a**) BTESEgU homopolymer membrane, and (**b**) BHETESPU-BTESPA (1:4), (**c**) BTESEgU-BTESPA (1:3), and (**d**) TTESPUEA-BTESPA (1:7) copolymer membranes. The bright spots on the surface appear to be due to contaminants, such as dust. In the cross-section images, the lower part corresponds to the PSQ layer.

The RO performance of these membranes depended on both the monomer structures and their ratios. The concentrations of the sols used to coat the support membranes also affected the RO performance of the membranes, and the effects were investigated using BTESEgU-BTESPA (1:1) as an example. Decreasing the concentration from 1.0 to 0.5 wt% tended to increase the water permeability and decrease the NaCl rejection, along with the tradeoff relationship between these parameters, resulting in a well-balanced performance using 0.5 wt% sol. However, at a concentration of 2.5 wt% for BTESEgU-BTESPA (1:1), both parameters decreased, likely due to the exceedingly enhanced aggregate formation. Therefore, all other membranes were prepared using 0.5 wt% sols for coating. In all cases, the homopolymerization of the urea-containing monomers provided membranes with low NaCl rejection, whereas copolymerization with BTESPA drastically improved the NaCl rejection. This was likely due to the aggregation of urea units in the homopolymer membranes, which generated defects in the PSQ layers of the membranes, as observed for HETESPU homopolymer membranes [24]. In fact, the SEM image of the BTESEgU homopolymer membrane surface revealed cracks on its surface (Figure 8a). It was also found that the copolymer membranes showed much superior performance compared to the BTESPA homopolymer membrane (water permeability = 1.1 × 10^−13^ m^3^ m^−2^s^−1^Pa^−1^ and NaCl rejection = 96%) [36]. Increasing the BTESPA ratio in the copolymers tended to improve both parameters and then to decrease them, giving optimized ratios of 1:4, 1:3, and 1:7 for the BHETESPU-BTESPA, BTESEgU-BTESPA, and TTESPUEA-BTESPA membranes, respectively. For the membranes exhibiting optimal performance, RO experiments were repeated three or four times (Figure 7b), and the best-balanced data are shown in Table 2 and Figure 7a. As shown in Figure 7b, the effects of the monomer structure and monomer ratio were significantly greater than the experimental error. These membranes exhibited the RO performance, comparable to that of the HETESPU-BTEAPA (1:3) membrane (Table 2) [24], with a slightly lower water permeability and a slightly higher NaCl rejection along the tradeoff relationship of these parameters. The superior performance of the BHETESPU-BTESE (1:4) and BTESEgU-BTESPA (1:3) membranes to the TTESPUEA-BTESPA (1:7) membrane is likely due to the higher hydrophilicity of BHETESPU and BTESEgU containing hydroxy and ethylene glycol units, respectively, than that of TTESPUEA. The lower hydrophilicity of TTESPUEA required a higher proportion of BTESPA to achieve the optimal performance of the TTESPUEA-BTESPA membrane, compared to the BHETESPU-BTESPA and BTESEgU-BTESPA membranes.

The SEM surface and cross-section images of the copolymer membranes are presented in Figure 8b–d. For the BTESEgU-BTESPA (1:3) and TTESPUEA-BTESPA (1:7) copolymer membranes, indentations with a maximum diameter of approximately 1 μm were observed on the surface, presumably due to the shrinkage of the PSQ structures through the enhanced network formation and urea hydrogen bonding (Figure 8c,d). The formation of indentations was more pronounced in the TTESPUEA-BTESPA (1:7) copolymer membrane. It is probable that the indentations did not fully penetrate the PSQ layer, judging from the relatively high NaCl rejections of these membranes. However, this seems to be partially responsible for the relatively low NaCl rejections of these membranes compared to the BHETESPU-BTESPA (1:4) membrane. The SEM image of the BHETESPU-BTESPA (1:4) membrane shows a smooth and uniform surface without indentations. The balance between the tendency to form aggregates and the hydrophilicity of urea-based organic units seems to be important for the good performance of the membranes. As previously mentioned, the lower hydrophilicity of TTESPUEA is the reason why a higher ratio of BTESPA is required to achieve the highest performance compared to BHETESPU and BTESEgU. In addition, TTESPUEA should have the highest tendency to enhance the aggregate and network formation, as its structure contains a larger number of urea and triethoxysilyl units, thereby requiring a higher ratio of BTESPA.

### 3.3. Chlorine and Heat Resistance

**Figure 9:**
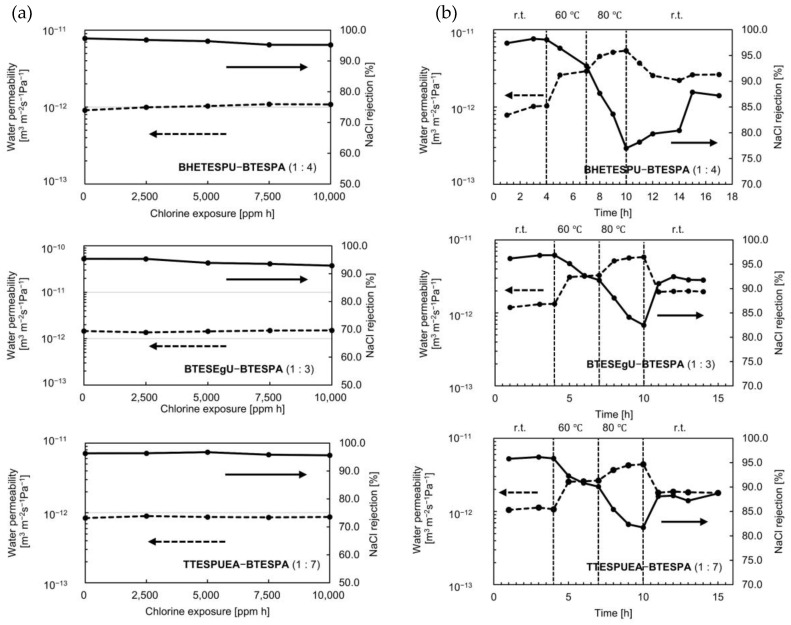
Changes in RO performance of urea-containing membranes upon (**a**) exposure to chlorine and (**b**) operation at elevated temperatures.

The chlorine resistance of the membranes was examined by immersing them in a 1000 ppm NaOCl aqueous solution at room temperature. As shown in Figure 9a, the membranes exhibited high chlorine resistance, as expected. They showed only 1–3% decreases in NaCl rejection, even after 10,000 ppm h exposure to chlorine, together with 3–19% increases in water permeability. This contrasted with the HETESPU-BTESPA membranes, which underwent evident deterioration under the same conditions, reducing the NaCl rejection by approximately 20% from the original data prior to exposure to chlorine [24]. This is likely due to the reduced number of NH bonds in BHETESPU and the bridged structures in BTESEgU and TTESPUEA. Bridged structures can prevent the disassembly of network PSQ structures, even if the urea units are partially decomposed. The heat resistance of the membranes was examined by conducting RO experiments at high temperatures. Increasing the operational temperatures resulted in enhanced water permeability and a reduction in NaCl rejection, likely due to the thermally enhanced vibration of the PSQ network. Upon conducting the RO experiments at 60 °C and subsequently cooling to room temperature, the original data were recovered. However, once the operation temperature was raised to 80 °C, the original data could not be recovered by cooling the temperature to room temperature. The water permeability increased and the NaCl rejection decreased by changing the temperatures, as shown in Figure 9b. This indicates that the membrane partially deteriorated at 80 °C, similar to the HETESPU-BTESPA membranes [24]. Among them, the BTESEgU-BTESPA (1:3) membrane exhibited the highest heat resistance, exhibiting only a 4.5% decrease in NaCl rejection. This is similar to the HETESPU-BTESPA (1:3) membrane, which showed a 4.9% decrease in NaCl rejection under the same conditions (from 96.1% to 91.2%) [24].

## 4. Conclusions

In summary, three new urea-containing triethoxysilanes were prepared, and they were found to be suitable monomers for the preparation of PSQ-based robust RO membranes for water desalination. As expected, these urea-containing membranes exhibited good performance as RO membranes, comparable to that of previously reported HETESPU-BTESPA copolymer membranes [24]. Additionally, it was demonstrated that reducing the number of chlorine-sensitive NH bonds and enhancing the network structure of PSQ are effective methods for improving the chlorine resistance of the resulting membranes. Notably, the examined membranes showed only 1–3% decreases in NaCl rejection, even after 10,000 ppm h exposure to chlorine, together with 3–19% increases in water permeability. Chemical stability, such as chlorine resistance, is critical to expand the membrane lifespans and to enable the use of a wide range of feed waters [37]. These monomers are readily accessible via one-step reactions using commercially available starting materials. This study provides a new molecular design for robust and high-performance RO membranes that can be prepared through a simple sol–gel process. They do not require additional nanofillers or composite formation [9,10,11,12,13,14,15,38]. Studies on further improvement of the performance of urea-containing RO membranes by employing comonomers other than BTESPA and the use of these urea-containing triethoxysilanes as precursors for other PSQ-based materials are underway, and the results will be reported elsewhere.

## Figures and Tables

**Table 1:** Sol formation by copolymerization of BHETESPU, BTESEgU, and TTESUEA with BTESPA.

Monomer /g (/mmol)	BTESPA /g (/mmol)	Ethanol /g	Water /g (/mmol)	Time /h
BHETESPU				
0.100 (0.287)	0	1.440	0.460 (25.52)	2
0.075 (0.213)	0.225 (0.529)	4.899	0.801 (44.45)	3
0.050 (0.142)	0.200 (0.470)	4.089	0.661 (36.68)	2
0.025 (0.071)	0.125 (0.294)	2.456	0.394 (21.86)	3
0.025 (0.071)	0.225 (0.528)	4.102	0.648 (36.00)	2
BTESEgU				
0.150 (0.233)	0	2.598	0.252 (14.00)	8
0.100 (0.156)	0.100(0.235)	3.378	0.422 (23.42)	3
0.050 (0.078)	0.150 (0.352)	3.335	0.465 (25.80)	3
0.050 (0.078)	0.200 (0.470)	4.158	0.592 (32.85)	2
TTESPUEA				
0.100 (0.113)	0	1.717	0.183 (10.16)	8
0.050 (0.056)	0.150 (0.352)	3.358	0.442 (24.53)	2
0.050 (0.056)	0.200 (0.470)	4.181	0.569 (31.58)	2
0.025 (0.028)	0.150 (0.352)	2.914	0.411 (22.81)	2
0.025 (0.028)	0.175 (0.411)	3.325	0.475 (26.36)	2
0.025 (0.028)	0.225 (0.528)	4.148	0.602 (33.41)	2

**Table 2:** RO performance of the urea-containing PSQ membranes.

**Monomer**	**BTESPA**	**sol Conc**	**Water Permeability**	**NaCl**
	**/Monomer**	**/wt%**	**/** **10^−13^ m^3^ m^−2^s^−1^Pa^−1^**	**Rejection/%**
BHETESPU	0	0.5	56.6	63.5
	3	0.5	12.7	94.0
	4	0.5	10.2	98.2
	5	0.5	11.3	92.6
	9	0.5	10.5	89.4
BTESEgU	0	0.5	18.8	48.9
	0	2.5	5.69	78.5
	1	0.5	11.4	89.1
	1	1.0	3.70	90.7
	1	2.5	6.68	76.5
	3	0.5	15.2	96.4
	3	1.0	2.89	92.0
	4	0.5	14.4	84.8
TTESPUEA	0	0.5	28.5	73.6
	3	0.5	13.5	88.4
	4	0.5	13.0	84.4
	6	0.5	12.3	91.0
	7	0.5	11.3	96.2
	9	0.5	9.74	92.0
HETESPU	3	0.5	18.6	95.9

## Data Availability

Data is contained within the article.

## References

[1] Sholl D.S., Lively R.P. (2016). Seven chemical separations to change the world. Nature.

[2] Li D., Wang H. (2010). Recent developments in reverse osmosis desalination membranes. J. Mater. Chem..

[3] Li D., Yan Y., Wang H. (2016). Recent advances in polymer and polymer composite membranes for reverse and forward osmosis processes. Prog. Polym. Sci..

[4] Hailemariam R.H., Woo Y.C., Damtie M.M., Kim B.C., Park K.-D., Choi J.-S. (2020). Reverse osmosis membrane fabrication and modification technologies and future trends: A review. Adv. Colloid Interface Sci..

[5] Goh P.S., Ismail A.F. (2020). Chemically functionalized polyamide thin film composite membranes: The art of chemistry. Desalination.

[6] Zuo H.-R., Shi P., Duan M. (2020). A review on thermally stable membranes for water treatment: Material, fabrication, and application. Serp. Purif. Technol..

[7] Kang G.-D., Gao C.-J., Chen W.-D., Jie X.-M., Cao Y.-M., Yuan Q. (2007). Study on hypochlorite degradation of aromatic polyamide reverse osmosis membrane. J. Membr. Sci..

[8] Liu M., Wu D., Yu S., Gao C. (2009). Influence of the polyacyl chloride structure on the reverse osmosis performance, surface properties and chlorine stability of the thin-film composite polyamide membranes. J. Membr. Sci..

[9] Xu G.-R., Wang J.-N., Li C.-J. (2013). Strategies for improving the performance of the polyamide thin film composite (PA-TFC) reverse osmosis (RO) membranes: Surface modifications and nanoparticles incorporations. Desalination.

[10] Liu Y., Xin Z., Wang M., Wang X., Zhang H., Wang Z. (2024). Optimizing separation layer structure of polyamide composite membrane for high permselectivity based on post-treatment: A review. Desalination.

[11] Shao F., Dong L., Dong H., Zhang Q., Zhao M., Yu L., Pang B., Chen Y. (2017). Graphene oxide modified polyamide reverse osmosis membranes with enhanced chlorine resistance. J. Membr. Sci..

[12] Gohil J.M., Suresh A.K. (2017). Chlorine attack on reverse osmosis membranes: Mechanisms and mitigation strategies. J. Membr. Sci..

[13] Khorshidi B., Biswas I., Ghosh T., Thundat T., Sadrzadeh M. (2018). Robust fabrication of thin film polyamide-TiO_2_ nanocomposite membranes with enhanced thermal stability and anti-biofouling propensity. Sci. Rep..

[14] He M., Wang L., Zhang Z., Zhang Y., Zhu J., Wang X., Lv Y., Miao R. (2020). Stable Forward Osmosis Nanocomposite Membrane Doped with Sulfonated Graphene Oxide@Metal–Organic Frameworks for Heavy Metal Removal. ACS Appl. Mater. Interfaces.

[15] Matshetshe K., Sikhwivhilu K., Ndlovu G., Tetyana P., Moloto N., Tetana Z. (2022). Antifouling and antibacterial β-cyclodextrin decorated graphene oxide/polyamide thin-film nanocomposite reverse osmosis membranes for desalination applications. Sep. Purif. Technol..

[16] Chang C.-M., Zhao Q., Chen S.B. (2025). Solvent-assisted insertion of molecular supports for enhanced separation performance and stability of thin film composite reverse osmosis membranes. J. Membr. Sci..

[17] Abe Y., Gunji T. (2004). Oligo- and polysiloxanes. Prog. Polym. Sci..

[18] Gon M., Tanaka K., Chujo Y. (2018). Recent progress in the development of advanced element-block materials. Polym. J..

[19] Du Y., Liu H. (2020). Cage-like silsesquioxanes-based hybrid materials. Dalton Trans..

[20] Kim J., Park Y., Kwon M.S. (2024). Recent progress in ladder-like polysilsesquioxane: Synthesis and applications. Mater. Chem. Front..

[21] Yamamoto K., Ohshita J. (2019). Bridged polysilsesquioxane membranes for water desalination. Polym. J..

[22] Zhang D., Kanezashi M., Tsuru T., Yamamoto K., Gunji T., Adachi Y., Ohshita J. (2022). Development of PSQ-RO membranes with high water permeability by copolymerization of bis [3-(triethoxysilyl)propyl]amine and triethoxy(3-glycidyloxypropyl)silane. J. Membr. Sci..

[23] Zhang D., Kanezashi M., Tsuru T., Yamamoto K., Gunji T., Adachi Y., Ohshita J. (2023). Preparation of thermally stable 3-glycidyloxypropyl-POSS-derived polysilsesquioxane RO membranes for water desalination. J. Membr. Sci..

[24] Zhang D., Kanezashi M., Tsuru T., Yamamoto K., Gunji T., Adachi Y., Ohshita J. (2022). Development of Highly Water-Permeable Robust PSQ-Based RO Membranes by Introducing Hydroxyethylurea-Based Hydrophilic Water Channels. ACS Appl. Mater. Interfaces.

[25] Yamamoto K., Amaike Y., Tani M., Saito I., Kozuma T., Kaneko Y., Gunji T. (2022). Bridged organosilica membranes incorporating, carboxyl-functionalized cage silsesquioxanes for water desalination. J. Sol-Gel Sci. Technol..

[26] Yamamoto K. (2022). Development of reverse osmosis membranes by incorporating polyhedral oligomeric silsesquioxanes (POSSs). Polym. J..

[27] Xu R., Cheng S., Cheng X., Qi L., Zhong J., Liu G.P., Huang M., Wasnik P., Jiang Q. (2023). In situ carboxyl functionalization of hybrid organosilica reverse osmosis membranes for water desalination. Adv. Compos. Hybrid Mater..

[28] Kamitani T., Ishida A., Imoto H., Naka K. (2022). Supramolecular organogel of polyureas containing POSS units in the main chain: Dependence on POSS and co-monomer structures. Polym. J..

[29] Takeuchi J., Tokuami I., Sakurai S., Imoto H., Naka K. (2025). Formation of supramolecular gels by self-assembly of dumbbell-shaped polyhedral oligomeric silsesquioxane derivatives linked with bisurea groups. Bull. Soc. Chem. Jpn..

[30] Hatakeyama E.S., Gabriel C.J., Wiesenauer B.R., Lohr J.L., Zhou M., Noble R.D., Gin D.L. (2011). Water filtration performance of a lyotropic liquid crystal polymer membrane with uniform, sub-1-nm pores. J. Membr. Sci..

[31] Niimi T., Nagasawa H., Kanezashi M., Yoshioka T., Ito K., Tsuru T. (2014). Preparation of BTESE-derived organosilica membranes for catalytic membrane reactors of methylcyclohexane dehydrogenation. J. Membr. Sci..

[32] Blatchley E.R., Chen M. (2010). Reaction Mechanism for Chlorination of Urea. Environ. Sci. Technol..

[33] Bergsman D.S., Closser R.G., Tassone C.J., Clemens B.M., Nordlund D., Bent S.F. (2017). Effect of Backbone Chemistry on the Structure of Polyurea Films Deposited by Molecular Layer Deposition. Chem. Mater..

[34] Schaber P.M., Colson T., Higgins S., Thielen D., Anspach B., Brauer J. (2004). Thermal Decomposition (Pyrolysis) of Urea in an Open Reaction Vessel. Thermochim. Acta.

[35] Horata K., Yoshio T., Miyazaki R., Adachi Y., Kanezashi M., Tsuru T., Ohshita J. (2024). Preparation of Polysilsesquioxane-based CO_2_ Separation Membranes with Thermally Degradable Succinic Anhydride and Urea Units. Separations.

[36] Yamamoto K., Koge S., Sasahara K., Mizumo T., Kaneko Y., Kanezashi M., Tsuru T., Ohshita J. (2017). Preparation of bridged polysilsesquioxane membranes from bis [3-(triethoxysilyl)propyl]amine for water desalination. Bull. Chem. Soc. Jpn..

[37] Lim Y.J., Goh K., Nadzri N., Wang R. (2025). Thin-film composite (TFC) membranes for sustainable desalination and water reuse: A perspective. Desalination.

[38] Zhang D., Kanezashi M., Tsuru T., Yamamoto K., Gunji T., Adachi Y., Ohshita J. (2022). Development of robust and high-performance polysilsesquioxane reverse osmosis membranes modified by SiO_2_ nanoparticles for water desalination. Sep. Purif. Technol..

